# Endoscopic Retrieval of Ingested Foreign Bodies: A Single Surgeon Experience

**DOI:** 10.7759/cureus.19293

**Published:** 2021-11-05

**Authors:** Nandkishor Sopanrao Sude, Venkata Pavan Kumar Karanam

**Affiliations:** 1 General Surgery, Employees State Insurance Corporation Medical College and Hospital, Hyderabad, IND

**Keywords:** upper gi tract, ingested, retrieval, foreign body, endoscopy

## Abstract

Introduction

Foreign body (FB) ingestion either accidental or intentional is a common clinical scenario encountered by general surgeons. This article reports a single surgeon's experience of endoscopic retrieval of foreign bodies from the upper gastrointestinal (UGI) tract.

Methods

A retrospective analysis of data of all the patients who underwent endoscopic management for foreign body removal by a single surgeon in a tertiary care hospital in southern India between 2015 and 2020 was conducted. Patient variables in terms of age, sex, type of foreign body, its location in the gastrointestinal (GI) tract, the time between ingestion and presentation, the time between presentation and endoscopy, treatment outcomes were reviewed.

Results

A total of 97 patients were studied. The age range of the patients studied was one month to 71 years. Males were predominant (n=64, 65.97%). The most common retrieved foreign body were coins (n=31, 31.9%). The most common site of foreign body lodgment was the esophagus (n=75, 77.31%), with the upper third esophagus (n=38; 39.37%) being the predominant site. The success rate of endoscopic retrieval in our study was 97%. No procedure-related complications were encountered in any patient. Endoscopic management failed in two patients who ultimately required surgical intervention.

Conclusion

Endoscopic retrieval of foreign bodies in the UGI tract is a safe and effective modality. Early endoscopy in such patients avoids surgical intervention and reduces morbidity.

## Introduction

An ingested foreign body (FB) is a common clinical scenario encountered in the surgical emergency department. Accidental ingestion is common in the extremes of ages. Pediatric patients, mainly infants, toddlers, and preschoolers, are at a higher risk for FB ingestion, the reason being curiosity typical of the development of infants, toddlers, and preschoolers, their tendency to put everything in the mouth, swallowing coordination difficulties due to immaturity [1]. Elderly persons often ingest foreign bodies because of impaired swallowing controls and intraoral sensitivity [2]. In adults, food bolus impaction due to underlying esophageal pathology is a common condition requiring endoscopic intervention. Intentional non-food foreign body ingestion in adults is mainly due to underlying psychiatric disorders, drug abuse, or suicidal tendencies [3].

The spectrum of foreign bodies ingested consists of blunt objects-round objects (coin, button, rings, bottle caps), sharp-pointed objects-fine objects (needle, safety pin, bone), sharp irregular objects (partial dentures), long objects-soft objects (wires), hard objects (toothbrush), food bolus, bezoars, objects containing chemicals (button batteries) [4]. Most of the ingested foreign bodies (80-90%) pass spontaneously. However, approximately 10% to 20% of foreign bodies require an endoscopic procedure for removal, and <1% require surgery [5]. Impacted foreign bodies at natural constriction sites in the upper gastrointestinal (UGI) tract, sharp objects piercing the wall, objects containing chemicals (button batteries) can lead to complications like obstruction, perforation, and corrosive injuries, respectively.

Early flexible UGI endoscopic examination helps in the effective retrieval of foreign bodies and prevents complications and the need for surgical intervention. The aim of this study is to report the experience of a single surgeon in the endoscopic management of foreign bodies in the UGI tract.

## Materials and methods

A retrospective analysis of the data from electronic medical records of all patients who underwent endoscopic management for foreign body removal by a single surgeon in a tertiary care hospital in southern India between 2015 and 2020 was conducted. A total of 97 patients were enrolled in this study. This study was conducted in adherence to the declaration of Helsinki.

Pre-procedure preparation

In this study, all the patients were initially examined by a general surgeon experienced in gastrointestinal endoscopy. Presenting symptoms and circumstances of foreign body ingestion were noted. In the case of children, detailed evaluation in respect of symptoms and clinical examination was done with the assistance of parents. A preliminary radiological assessment using a plain X-ray was done for all patients to assess the probable location of the FB lodgment. Patients who presented to us along with X-rays done elsewhere were not subjected to a repeat radiological investigation. After confirmation of the probable location of the foreign body, the patients and their parents (in the case of children) were counselled regarding the type of management, the procedure of endoscopy, and complications. In some children, although the foreign bodies were deemed passable without an intervention, endoscopy was done at the insistence of apprehensive parents. After obtaining valid informed consent, patients were shifted for endoscopy. All the endoscopic upper GI tract foreign body retrievals were done as emergency procedures.

Endoscopic procedures and settings

Airway protection is of special concern during foreign body removal from the UGI tract. All the children and most of the adults in our study underwent the procedure under general anesthesia with endotracheal intubation in order to prevent the inadvertent transfer of the foreign body into the airway during the retrieval procedure and to prevent aspiration especially in patients with a full stomach, proximal esophageal location of the foreign body, food bolus impaction. In a small number of adult patients who did not consent for general anesthesia, the procedure was performed under local pharyngeal anesthesia after explaining the possible risks and the retrieval was done with utmost care. Foreign body removal without endotracheal intubation can be performed with the use of the latest devices like overtubes that extend past the upper esophageal sphincter which not only protect the airways but also facilitate passage of the endoscope during removal of foreign body. The unavailability of such modern devices was a major limitation of our study.

After airway intubation, a flexible endoscope (EG-530WR; Fujifilm Corporation, Tokyo, Japan) was inserted perorally through the mouth-gag. After visualization and assessment of the nature of the foreign body in terms of size, shape, and edges, appropriate retrieval devices were selected. Generally, biopsy forceps, graspers, rat tooth forceps were employed for linear, sharp-pointed foreign bodies. For blunt or irregular sharp-pointed foreign bodies, biopsy forceps, graspers, polypectomy snares, or baskets were used. If a retrieval device failed to grasp the foreign body, another type of device was employed for retrieval. If the retrieval procedure was expected to cause major injury to the upper GI tract, the endoscopy was abandoned. After successful retrieval of foreign bodies, a repeat endoscopy was performed to examine for any remnants of the foreign body and to rule out injuries of the UGI tract. In some cases, fluoroscopy using C-arm was performed for confirming successful retrieval. Post procedure all the patients were observed for a day and advised for discharge once they were fit. Patients with psychiatric illness were referred to the psychiatry department for counselling and treatment.

Data collection

In this study, clinical variables, such as age, sex, type of foreign body, its location in the gastrointestinal tract, the time between ingestion and presentation, the time between presentation and endoscopy, and treatment outcomes were analyzed

Statistical analysis

The data were analyzed using IBM SPSS Statistics for Windows, Version 27.0 (Armonk, NY: IBM Corp.), and descriptive statistics were expressed as a number and a percentage for categorical variables and as mean standard deviation for quantitative data.

## Results

Patient characteristics and clinical presentations

The age range of the patients studied was one month to 71 years. The distribution of the patients according to their age was skewed. Hence, the mean and standard deviation for children and adults were calculated separately. The majority of patients were found to be less than 14 years of age. The mean (±SD) age of the children (< 14 years) who received endoscopic management of foreign bodies was 5 (±3) years. The mean (±SD) age of the adults who received endoscopic management of foreign bodies was 48 (±20) years. Most of the patients were of the age group 1-10 years (n=68; 70.1%) followed by infants (n=15; 15.46%; Table 1). Male patients predominated in our study (n = 64; 65.97%). Common clinical symptoms observed were sudden onset dysphagia, pain in the throat, foreign body sensation in adults and drooling, throat pain, and vomiting in children.

**Table 1:** Patient characteristics

Age groups (years)	Male n(%)	Female n(%)	Total n (%)
0-1	8 (8.24)	7 (7.21)	15 (15.46)
1-10	44 (45.36)	24 (24.74)	68 (70.1)
10-20	2 (2.06)	1 (1.03)	3 (3.09)
20-40	4 (4.12)	0	4 (4.12)
40-50	0	0	0
50-60	3 (3.09)	0	3 (3.09)
>60	3 (3.09)	1 (1.03)	4 (4.12)
Total	64 (65.97)	33 (34.02)	97 (100)

Locations of foreign bodies

The most common site of foreign body lodgment was the esophagus (n =75; 77.31%), with the upper one third esophagus (n = 38; 39.17%) being the predominant site. Other lodgment sites were the middle third esophagus (n = 25; 25.77%), stomach (n=20; 20.61%), lower third esophagus (n = 12; 12.37%), and duodenum (n = 2; 2.06%; Table 2).

**Table 2:** Location of foreign bodies

Location	Number of patients	Percentage (%)
Esophagus	75	77.31
Upper 1/3	38	39.17
Middle 1/3	25	25.77
Lower 1/3	12	12.37
Stomach	20	20.61
Duodenum	2	2.06
Total	97	100

Types of foreign bodies

**Figure 1:**
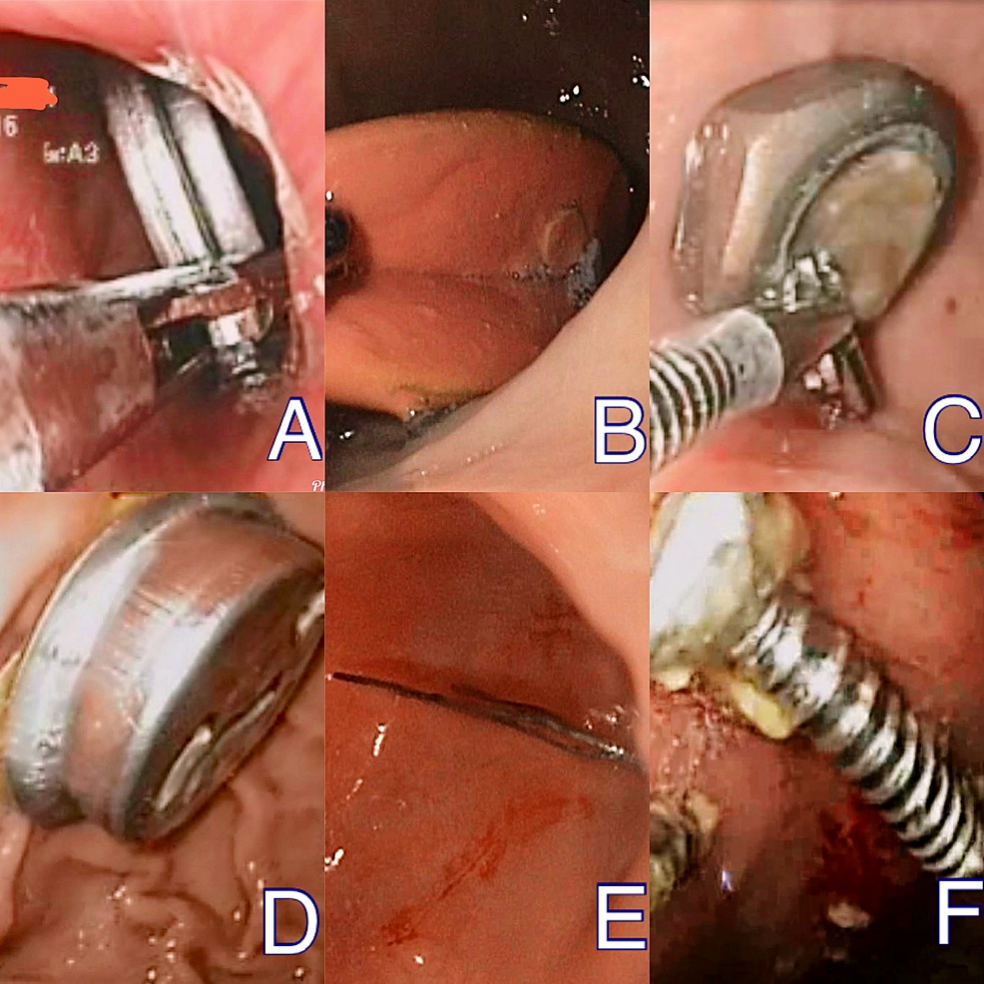
Various types of foreign bodies encountered in our study. (A) Coin, (B) plastic object, (C) metal rings, (D) steel cup, (E) hairpin, and (F) nail.

The major types of foreign bodies were blunt objects with metallic coins (Figure 1A) being most common (n=31; 31.9%) followed by blunt plastic objects (Figure 1B). Other types of foreign bodies included: metal rings (Figure 1C), steel cups (Figure 1D), sharp objects like hairpins (Figure 1E), nails (Figure 1F), fish bones, dentures, electric wires, toothbrushes, and button batteries. The distribution of types of foreign bodies is shown in Table 3.

**Table 3:** Types of foreign bodies

Type	Number of patients	percentage
Blunt objects (metallic)		
Coins	31	31.9
Rings	12	12.37
Blunt objects (non-metallic)		
Bottle caps	5	5.15
Plastic objects	16	16.49
Sharp pointed objects		
Pins	6	6.18
Fishbone	5	5.15
Sharp irregular objects		
Dentures	4	4.12
Long soft objects		
Electrical wires	1	1.03
Long hard objects		
Toothbrush	1	1.03
Food bolus	3	3.09
Objects containing chemicals		
Button batteries	5	5.15
Others	8	8.24
Total	97	100

Timing of endoscopic management

The mean time of presentation to our hospital after ingestion of foreign body was 23.1 (±2.6) hours. The mean time between presentation to hospital and endoscopy was 6.8 (±3.2) hours. Patients with a history of ingestion of sharp objects and button batteries underwent endoscopy within six hours.

Endoscopy outcomes

Endoscopic retrieval was successful in all the patients except two patients. There were no procedure-related complications in the patients who underwent endoscopic retrieval. In two patients, a cervical esophagotomy was done to remove the foreign body. Both the patients were aged above 60 years and both accidentally ingested dentures which were lodged in the upper one-third of the esophagus. The success rate of endoscopic retrieval in our study was 97%.

## Discussion

Foreign body ingestion is a common surgical emergency encountered in the emergency department and a timely endoscopic intervention can prevent disastrous consequences. A skilled endoscopist can retrieve most of the foreign bodies successfully with minimal complications [6].

Accidental foreign body ingestion is frequently encountered in the pediatric population (85.46%). Infants, toddlers, and preschoolers are at higher risk [7]. Consistent with the available literature, most of the patients in our study belonged to these groups of children, owing to their tendency to put everything in their mouths out of curiosity. Most of the middle-aged adults in our study ingested foreign bodies intentionally due to psychiatric disorders. Four patients aged more than 60 years accidentally ingested dentures due to impaired swallowing controls [8].

Most of the ingested foreign bodies get lodged at the four physiologically narrow sites of the esophagus: the upper esophageal sphincter, level of the aortic arch, main stem bronchus, and lower esophageal sphincter [9]. In our observation, most of the foreign bodies were lodged in these physiologically narrow sites of the esophagus (77.31%) without any underlying pathology, predominantly in the upper third (39.17%). These findings are consistent with large series of studies conducted by Li et al. [10] and Zhang et al. [11]. Foreign bodies lodged in the esophagus generally present with symptoms like crying with drooling, throat pain in children and are frequently noticed by parents. Sudden onset dysphagia and odynophagia are symptoms noticed in adults [12]. Objects less than 2 cm traversed the esophagus and were lodged in the stomach (20.61%) and the duodenum (2.06%) [13]. Although patients with foreign bodies in these locations were asymptomatic and such foreign bodies were deemed passable without intervention, endoscopy was done because of the insistence of apprehensive parents or the foreign bodies being sharp objects or objects containing toxins (batteries).

**Figure 2:**
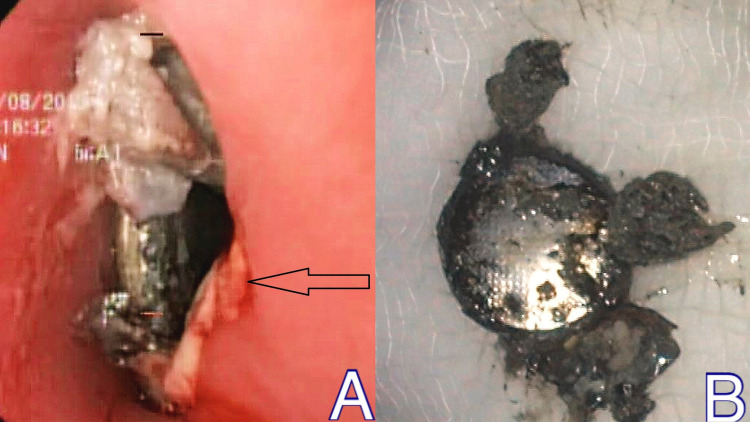
Button battery ingestion (A) Endoscopic view with an arrow pointing esophageal mucosal erosions and (B) retrieved button battery

**Figure 3:**
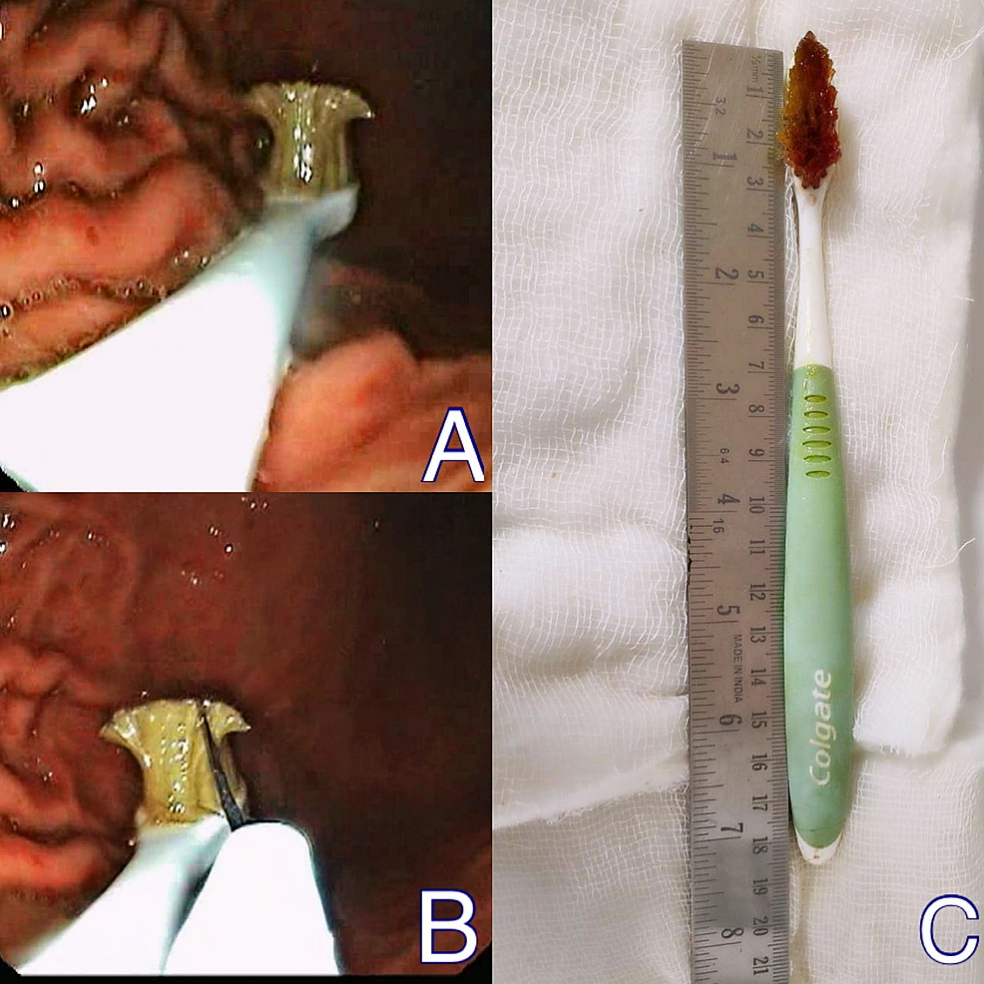
Toothbrush ingestion (A) Endoscopic view of stomach showing toothbrush, (B) snare passed over brush, and (C) retrieved toothbrush

Blunt round objects (49.42%) were the commonly retrieved foreign bodies in our study with coins (31.9%) being the most common. Coins are the most commonly found foreign bodies in many studies worldwide [14,15]. Commonly encountered foreign bodies in the pediatric population are coins, small metal rings, bottle caps, plastic toys, and button batteries as these are easily available within their reach [16]. Most of these were parts of toys or improperly disposed objects. Button batteries, when ingested, become lodged in the upper esophagus and react quickly with saliva. The battery discharges a current that hydrolyses water and generates hydroxide, creating a caustic (alkaline) injury to the tissue. In one child who ingested a button battery, esophageal mucosal erosions were observed due to caustic injury (Figure 2A-2B). Food bolus and fishbone impaction are common reasons requiring endoscopic intervention in middle-aged adults [17]. One young male patient with psychiatric illness intentionally swallowed an 18 cm long toothbrush which was retrieved using a snare (Figure 3A-3C). Accidentally ingested dentures in the old-aged patients were found impacted in the upper esophagus. These were partial dentures with irregular sharp borders.

Plain radiography of the neck or chest was helpful in detecting the presence of as well as assessing the location, size, configuration, and the number of ingested foreign bodies in our study [18]. These findings were helpful for better planning of the endoscopic intervention. Since most of the foreign bodies were radio-opaque, the facility of real-time localization using C-arm guidance during the procedure can avoid routine CT scans and high radiation exposure.

European Society of Gastrointestinal Endoscopy (ESGE) recommends emergent (preferably within two hours, but at the latest within six hours) therapeutic esophagogastroduodenoscopy for foreign bodies inducing complete esophageal obstruction, and for sharp-pointed objects or batteries in the esophagus to reduce the risk of major complications such as perforation with or without mediastinitis, retropharyngeal abscess, and aortoesophageal fistula. Urgent (within 24 hours) therapeutic esophagogastroduodenoscopy is recommended for other esophageal foreign bodies without complete obstruction. The treatment outcome is significantly influenced by the timing of endoscopic intervention. In our study, the meantime of presentation to our tertiary care hospital after ingestion of foreign body was 23.1 (±2.6) hours. This delay in presentation is attributed to most patients being from far away rural areas, lack of skilled endoscopists in secondary care hospitals, and delay in referral. The mean time between presentation to hospital and endoscopy was 6.8 (±3.2) hours. In a study by Lee et al. [17], the “door to scope” time was 5.9 (±5.2) hours. Emergency endoscopy (within six hours) was done in patients with a history of ingestion of sharp objects and button batteries.

The choice of endoscopic method and equipment required for retrieval is generally based on the type and location of the ingested foreign bodies [19]. We employed either biopsy forceps, grasper, or rat tooth forceps for linear, sharp-pointed foreign bodies. For blunt or irregular sharp-pointed foreign bodies, biopsy forceps, graspers, polypectomy snares, or baskets were used (Video 1). The success rate of endoscopic retrieval in our study was 97% with zero procedure-related complications. Our results correlate with other studies. Endoscopic retrieval failed in two patients to remove impacted dentures as the dentures in these patients were partial dentures of a considerably bigger size. Endoscopy was abandoned midway and cervical esophagotomy was performed for extraction.

**Video 1:** Compilation of videos of retrieval of foreign bodies employing various devices.

Our study has some limitations. First, our study included patients of all age groups contrary to most of the studies in the literature which compared specific age groups. Another limitation was the lack of availability of advanced retrieval devices which can alter the outcome of the procedure. Due to zero procedure-related complications in our study, prospective studies with larger numbers of patients are required to assess the risk factors for foreign body removal-related complications in the UGI tract.

## Conclusions

Endoscopic retrieval of foreign bodies in the UGI tract is a safe and effective modality. Early endoscopy by a skilled endoscopist results in successful retrieval with minimal complications. A high index of suspicion is required in diagnosing foreign body ingestion in children presenting with symptoms like drooling. Flexible endoscopy should be the initial therapeutic modality in the algorithmic approach for ingested foreign bodies as it avoids surgical intervention and reduces morbidity.
